# Expression of cyclooxygenase-2 (COX-2) in colorectal carcinoma in an indigenous African population of Kano, Nigeria

**DOI:** 10.3332/ecancer.2024.1816

**Published:** 2024-12-06

**Authors:** Jimoh Ajanaku Abdulrazaq, Mohammed Aminu Zakari, Yusuf Ibrahim, Hamza Ahmad

**Affiliations:** 1 Department of Pathology, Federal University of Health Sciences Azare, Azare 751101, Bauchi, Nigeria; 2 Department of Pathology, Aminu Kano Teaching Hospital, Kano 700101, Nigeria; a https://orcid.org/0000-0003-4052-3300; b https://orcid.org/0000-0002-4545-866x; c https://orcid.org/0009-0004-4000-9285; d https://orcid.org/0009-0000-4078-7328

**Keywords:** COX-2 expression, colorectal cancer, indigenous Africa population, Kano, Nigeria

## Abstract

**Background:**

cyclooxygenases-2 (COX-2) over-expression has been noticed in colorectal cancers (CRCs) with adverse outcomes, serving as a potential marker for prognosis, targeted therapy and as a window in CRC prevention. Unfortunately, there are scarce data regarding COX-2 expression in CRC in Africa where CRC incidence is on the increase with younger age affectation and unfavourable outcomes.

**Aims:**

This retrospective study aims to determine the proportion of CRCs that over-express COX-2 and document any relationship between COX-2 over-expression with clinicopathological features such as histologic subtype, tumour grade, age and sex.

**Methods:**

All the 139 CRCs that were histologically diagnosed at Aminu Kano Teaching Hospital over a 5-year period were included, but only 124 Formalin-fixed paraffin-embedded tissue blocks were sectioned and stained with COX-2 antibody. COX-2 expression was scored for distribution (no cells = 0, 1%–10% = 1, 11%–50% = 2, 51%–80% = 3, 81%–100% = 4) and intensity (no stain = 0; weak = 1; moderate = 2, strong = 3). The immunoreactive score (IRS) is a product of intensity (I) and distribution (D) as: 9–12 strongly +, 5–8 moderately +, 1–4 weakly + and 0 negative. Over-expression of COX-2 is an IRS of 5–12. Outcomes were statistically evaluated with clinicopathological data.

**Results:**

The CRCs occurred more commonly in males (M: F, 2:1), in the middle age group (mostly between 30 and 59 years), and 51.1% of cases occurred before 50 years and peaked in the 6th decade. Over-expression of COX-2 was observed in 46.8% (58/124) and was strongly associated with adenocarcinoma (ADC) not otherwise specified (NOS) (moderately and poorly differentiated tumours) but not with age or sex.

**Conclusion:**

The over-expression of COX-2 was significantly associated with ADC NOS (moderately and poorly differentiated tumours), indicating that it may influence the outcome of CRCs with possible variation in tumour subtype.

## Introduction

Colorectal carcinoma (CRC) is a malignant epithelial tumour of the colon and rectum, and demonstrates a worldwide variation in incidence and outcomes [1, 2]. Though there are more cases in the Western world, the incidence is rising in Africa and Asia, and the majority of deaths occur in less-developed regions reflecting poorer survival in these regions [3, 4]. The available data from Africa has highlighted major challenges and these include rising incidence, attraction to the Western lifestyle, late presentation, younger age affectation, aggressive tumour sub-type, lack of tailored therapy, inadequate research, high cost of treatment, dearth of radiotherapy centres, lack of government support and poorer outcomes [5–7].

Expression of cyclooxygenases-2 (COX-2) in CRC is associated with adverse outcomes. For the pathological roles it plays and experimental observations, over-expression of COX-2 is associated with inflammation, initiation of cancer, cancer progression, activation of vascular endothelial growth factor, angiogenesis, proliferation of surviving cancer stem cells, invasion, metastasis and tumour relapse after therapy [8–10]. Genetically, the carriers of the COX-2 A-1195G AG genotype have 2 and 2.9 times increased risk of developing colorectal adenoma and cancer, respectively, while the respective risk of developing adenoma and CRC for COX-2 A-1195G AA genotype is 1.8 and 3.1 times lower than the general populace [11]. From epidemiological and clinical point of view, inhibitors of cyclooxygenases (COX1 and COX-2) such as nonsteroidal anti-inflammatory drugs (NSAIDs) are useful in the chemo-prevention and management of CRC [8–10]. Based on the sufficient data available, the use of NSAIDs has been recommended in USA and Australia for the dual benefits of cardiovascular events and cancer chemo-prevention [12, 13]. The burdens of CRC in Africa create the need for CRC prevention and treatment. One important way of preventing CRC is through the use of NSAIDs. Studies on the COX-2 expression in CRCs are limited in Africa as the majority of studies are mainly from Asian and Western Countries. This retrospective study aims to determine the proportion of CRCs that over-express COX-2 and document any relationship between COX-2 over-expression with clinicopathological features such as histologic subtype, tumour grade, age and sex.

## Materials and methods

**Figure 1.**
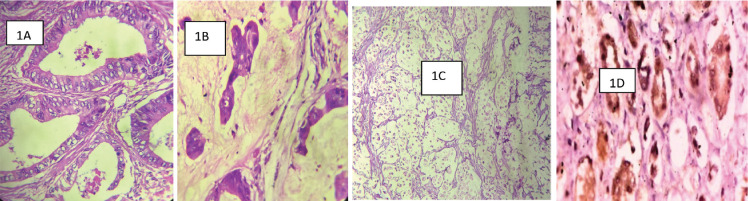
(Photomicrographs): (a): WDA of the colon showing well-formed malignant glands infiltrating muscularis propria. Haematoxylene and eosin (H&E) ×200. (b): A mucinous carcinoma of the rectum showing nests and cords of malignant epithelial cells surrounded by pools of mucin. H&E ×200. (c): A signet-ring cell carcinoma of the rectum showing signet-ring cell. H&E ×200. (d): COX-2 positive control. Typical cytoplasmic and membranous staining of COX-2 protein on the renal tubular epithelial cells ×200.

This retrospective hospital-based descriptive study was carried out on CRC cases histologically diagnosed in the Histopathology department of Aminu Kano Teaching Hospital, Kano (AKTH) in north-western Nigeria from 1st January 2015 to 31st December 2019. The hospital (AKTH) is a tertiary hospital with over 700-bed capacity. The histopathology department of AKTH receives an average of 5,500 histological samples per annum and renders services including cytology, histology, autopsy and immunohistochemistry (IHC). The CRCs cases were classified and graded according to World Health Organisation, WHO (2019, 5th edition) classification of tumours of the colon and rectum (Figure 1) [14]. The CRCs were grouped into adenocarcinoma (ADC) not otherwise specified (NOS) and other specific histological subtypes. The two-tiered system of grading was used on the basis of prognosis: Low-grade ADC which comprise of well-differentiated adenocarcinoma (WDA) and moderately differentiated adenocarcinoma (MDA) and high-grade ADC that is poorly differentiated adenocarcinoma (PDA) plus other specific sub-types [14]. Cases with insufficient clinical information, particularly biodata, missing or damaged blocks and tissue blocks with insufficient tissue were excluded.

Data, such as age, site of tumour, histologic diagnosis and grade of the disease was obtained from the Kano cancer registry, pathology request forms, patients’ case notes, duplicate copies of histopathological reports and slide reviews of cases. Ethical approval for this study was obtained from the Health and Research Ethics Committee of AKTH, Kano (ethical review reference number: AKTH/MAC/SUB/12A/P-3/V1/2904). The patient’s identity was concealed at all times and the additional consent for individuals whose samples were used in this study was waived by the institutional ethical committee.

### IHC for COX-2 expression

Immunohistochemical staining was performed using anti-COX-2 rabbit polyclonal antibody (Elabscience, USA, catalog No. E-AB-70031), used at a 1:500 dilution according to standard immunohistochemical staining protocols. The antibody was stored at −20 degrees Celsius and it was centrifuged before opening to ensure complete recovery of contents. A kidney sample with intact renal tubules was used as positive control while a negative control was obtained by replacing primary antibody with distilled water.

### Tissue preparations (FFPE tissue blocks)

Formalin-fixed paraffin-embedded tissues retrieved were cut or sectioned at 3 µm. The tissue slides were left to dry in an oven for 2 hours at 60º after microtomy. Then, the slides were dewaxed in xylene for 1 minute and then passed through decreasing concentrations of alcohol at 100%, 95% and 70% for 1 minute each for rehydration of sections.

### Antigen retrieval or unmasking of antigen sites

Antigen retrieval was done by placing the slides in 100 ml of citrate buffer (pH 6) and then heating using the microwave heating retrieval method at a hundred degrees centigrade (100^o^C) for 15–20 minutes. Slides were then washed in distilled water for another 2 minutes and then washed with phosphate buffer for 3 minutes twice. They were rinsed in distilled water for 2 minutes, and then 3% of hydrogen peroxide was then added and left for 10 minutes. Slides were then washed in running distilled water for 2 minutes and incubated with a blocking reagent for 10 minutes.

### Blockage of endogenous peroxidase

To block non-specific antigen sites, tissue sections were incubated for 1 hour in 1.5% bovine serum albumin at room temperature.

### Immunostaining procedure

Each slide was wiped with cotton wool to remove excess blocking solution. Incubation with the primary antibodies was carried out at room temperature for 30 minutes with 200 μl of anti-COX-2. Following the primary antibody incubation step, a secondary antibody from a streptavidin-biotin complex peroxidase kit (LSAB + kit, Dako, Copenhagen, Denmark) was then incubated according to the manufacturer’s instructions. Peroxidase activity was also developed with the substrate 3,3-diaminobenzidine tetrahydrochloride (DAB; Dako) by incubating the tissue sections in DAB for a period of 3 minutes. The tissue slides were washed in running water for a period of 3 minutes and then counterstained with haematoxylin. The tissue sections were then dehydrated in increasing concentrations of alcohol. They were cleared in xylene and the application of cover slip was done using Distyrene Plasticizer Xylene and they were allowed to dry.

**Figure 2.**
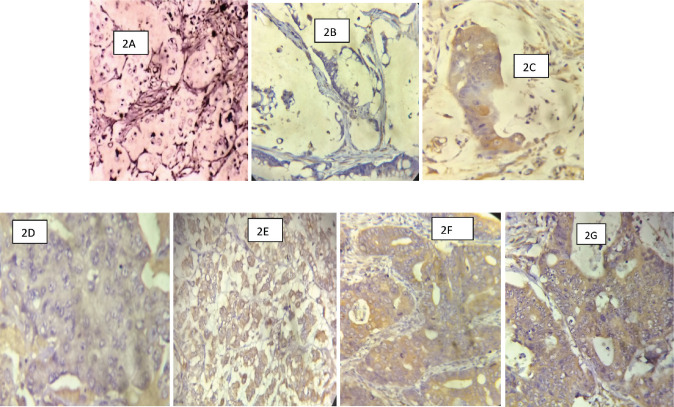
(Photomicrographs of CRCs with COX-2 IHC): (a): A signet-ring cell carcinoma of the colon showing no membranous/cytoplasmic brown staining (COX-2 negative) ×200. (b): A mucinous carcinoma of the rectum showing no membranous/cytoplasmic brown staining (COX-2 negative) ×200. (c): A mucinous carcinoma of the rectum showing strong membranous/cytoplasmic brown staining (COX-2 positive) ×200. (d): A PDA of the colon showing weak COX-2 stain ×200. (e): A PDA of the colon showing strong membranous/cytoplasmic brown staining (COX-2 positive) ×200. (f): A WDA of the colon showing strong membranous/cytoplasmic brown staining (COX-2 positive) ×200. (g): A MDA of the rectum showing strong membranous/cytoplasmic brown staining (COX-2 positive) ×200.

The slides were viewed under the light microscope, brown cytoplasmic and membranous staining was interpreted as positive staining and was scored semi-quantitatively using the immunoreactive score (IRS) system, a final score that is a product of the intensity and distribution of COX-2 immunoreactivity (Figure 2) [15]. The intensity of staining was scored as 0 for no staining, 1 for weak staining, 2 for moderate and 3 for strong staining. The percentage of positive tumour cells was scored: 0 indicating no cell with a positive reaction, 1 indicating 1%–10% of cells with a positive reaction, 2 indicating 11%–50% of cells with a positive reaction, 3 indicating 51%–80% of cells with a positive reaction and 4 indicating greater than 80% of cells with a positive reaction. The final IRS score obtained by multiplying the distribution and intensity for each tumour was graded as follows: 9–12 strongly positive, 5–8 moderately positive, 1–4 weakly positive and 0 negatives. COX-2 was considered over-expressed if the IRS score is moderate to strong (that is a score of 5 to 12), Table 1.

### Statistical analysis

Statistical analysis was performed using Statistical Package for Social Sciences (SPSS Inc, Chicago, IL, USA version 25). Chi-square was used with statistical significance set at *p* < 0.05 to test the association between COX-2 expression and clinico-pathological parameters such as age, sex, histological subtype and grade. Proportions and central tendencies of nominal and continuous variables were obtained by descriptive statistics of frequency, mean and median.

## Results

139 CRCs within the study period in the department were analysed and 124 cases had COX-2 IHC. Sixty-four CRC cases were endoscopic biopsies. Each of the 139 cases belongs to only one patient and there were no duplicate cases or combined open biopsies and endoscopic biopsies belonging to a single patient.

### Age distribution of CRCs

The age distribution of CRC cases ranged from 11 to 85 years with a mean age of 48.2 (S. D-15.5) years (Table 2). The largest proportion of cases (64.0%) clustered within the 30 to 59 age range and peaked in the 6th decade. Seventy-one cases of CRC (51.1%) occurred before the age of 50 years while only one case (0.7%) occurred at the age of 80 years or more. Out of 81 cases that occurred before or at 50 years, 30 cases (37.0%) occurred in females.

### Gender distribution of CRCs

There were 92 males and 47 females giving a male-to-female ratio of 2:1. Female cases tend to be associated with a younger age group than their male counterpart (mean of 45.7 years for females versus 49.5 years for males, and 63.8% of female cases occurring before or at 50 years versus 55.4%).

### Histological types of the CRCs

ADC NOS constitute 87.1% of cases (Table 2). Twelve (60.0%) of the ADC NOS with high-grade features were observed at ≤50 years and 40% of this occurred in females. Overall, 10 (83.3%) cases of mucinous carcinomas occurred at 50 years or below. Unfavourable subtypes such as poorly differentiated ADC NOS, mucinous carcinoma and signet ring cell carcinoma tend to affect younger age groups before or at 50 years (Table 2).

### Grades of the CRCs

Low-grade ADC made up 72.7% of cases (Tables 2 and 3). The respective proportions of low-grade and high-grade ADC that occurred at ≤50 years are 71.3% (72 of 101 cases) and 81.6% (31 of 38 cases).

### COX-2 IHC for CRCs

Over-expression of COX-2 was observed in 46.8% (58 out of 124) of cases. Of this number, 70.7% (41 of 58 positive cases) of this was moderately positive while 29.3% (17 of 58 positive cases) were strongly positive (Table 4).

### COX-2 expression and age of patients with CRCs

Out of 58 cases that over-expressed COX-2, 75.9% (44 of 58) occurred in the 30–69 age range, but it is interesting to note that no positivity was recorded in the age 10–19 and 80–89 categories which have 3 and 1 case, respectively (Table 5).

### COX-2 expression and sex of patients with CRCs

Forty-two out of 124 cases that had COX-2 IHC were females while the remaining 82 cases were males. Seventeen of 42 (40.5%) female cases were COX-2 positive which constitutes 29.3% of all COX-2 positive cases. Half of the male cases expressed COX-2 which accounted for the majority of COX-2 positive cases, 70.7% (41 of 58). The ratio of female to male cases that over-express COX-2 is 1: 2.4, but 40.5% of female and 50% of male cases expressed COX-2.

### COX-2 expression and histological sub-type of CRCs

For the category of ADC (NOS), 48.7% of cases (56 of 115) were positive and these include 33.3% of ADC with well-formed glands (17 of 51), 63.0% of ADC with moderate differentiation (29 of 46) and 55.6% of ADC with poor differentiation (10 of 18). Only 2 cases out of 8 (25%) cases of mucinous carcinomas over-expressed COX-2. COX-2 expression was strongly associated with histological sub-type (*p*-value-0.02101).

### COX-2 expression and grade of CRCs

Despite the fact that a large number of MDA tend to be positive compared to other sub-types (29 out of 46 cases, 63.0%), only 46 out of 97 (47.4%) cases of low-grade ADC (both well and MDA) over-expressed COX-2 (Table 5). Thirty-eight (27.3%) CRCs were high-grade ADC but only 12 out of 27 cases (44.44%) over-expressed COX-2.

## Discussion

### Age distribution of CRC

Early onset CRC: ‘Early-onset’ CRC refers generally to CRC occurring in patients ≤50 years old. This current study demonstrates that more than half (51.08%) of CRCs are early-onset. Similar findings have also been documented across Africa, India and Filipinos [5 ,16–24]. In Ibadan, Nigeria, 41% of cases occurred before 50 years; in Mali, 60% of cases were before 50 years; in Kenya, 17% of cases were observed before 40 years and in Ghana, 34% of cases manifested before 50 years [5, 16, 17, 19]. In Mumbai, India, Prachi et al showed the mean age of CRC at 47.2 years and 33% of cases were in those below 40 years [20]. Kaw *et al* [23] observed a mean age of 55.3 years and 17% of cases occurred in patients 40 years of age or younger in the Philippines [21]. In the United States of America, as a result of the rise in CRC among young individuals, the median age of CRC cases has declined from age 72 years in the early 2000s to age 66 years in 2018 [25]. The early onset CRC incidence is a global phenomenon, affecting USA, UK, Australia, Canada and Germany even though at a lower magnitude [26–28].

Certain birth cohorts have been identified as a possible cause of early onset CRC [29–31]. In this regard, a birth cohort effect results because age-specific CRC incidence rates vary due to changes in the ways by which generations are exposed to CRC risk factors. Those who were born in the 1950s have the lowest CRC incidence in the USA, but the risk of developing CRC has since increased with each successive generation [30]. Desk-bound work hours and passive media consumption independently increase the risk for young-onset CRC. In the USA, early onset CRC risk has increased by 69% for men and 20% for women for more than 14 hours per week spent watching television [32, 33]. A family history of CRC, [34–37] and certain key molecular alterations [38, 39] may also give an insight to the cause of early onset CRC.

## Sex distribution of CRC

Globally, incidence rates of CRC are higher in males than females. The current study demonstrated a male preponderance and sex (M: F) ratio of 2:1 which echoes the findings in other studies [5, 16, 20]. Contrastingly, a study from Filipinos showed no gender discrimination [23] while two studies from Ghana demonstrated higher female occurrence [19, 40]. The female preponderance in Raskin *et al* [19] (Ghana) and Kaw *et al* [23] (Philippines) may stem from the location of tumours as they observed more right-sided colonic tumours (with the exclusion of rectal tumours) which are more common in females compared to the left tumour. Differences in risk factor exposure and access to screening, hormonal factors and socioeconomic factors appear to be important in CRC sex disparity [41–44].

## Histological sub-types and grade of CRC

ADC NOS accounted for the majority (87.1%) including WDA and MDA, both of which accounted for 72.7% of all CRC and this finding is in consonance with other studies [5, 18, 19, 23]. However, the proportion (27.3%) of CRC with unfavourable histology put together is quite significant: 14.4% (PDA), 8.6% (mucinous carcinoma), 3.6% (signet-ring cell carcinoma) at 3.6% and 0.7% (clear cell carcinoma) which is similar to findings in Kenya, Ghana and Philippines [18, 19, 23].

## COX-2 expression in CRCs

Over-expression of COX-2 was observed in 46.8% of the CRCs in this study, which is in agreement with studies from Japan by Bamba *et al* [46], Konno* et al* [47], Tomozawa *et al* [48] and Yamauchi *et al* [49], and a study from the UK by Elder *et al* [45] where the rate of COX-2 over-expression ranged from 25% to 92%. The available studies demonstrated wide variation (25% to 92%) in COX-2 expression (Table 6). Konno *et al* [47] observed a rate of 25% of COX-2 positivity in Japan while Elder *et al* [45] demonstrated a 92% rate in the UK [45, 47]. Elder *et al* [45] examined the over-expression of COX-2 in 35 adenomas and 38 sporadic invasive colorectal ADC at the University of Bristol, UK, and showed that COX-2 expression in CRC was 92% compared to 66% in adenomas [45]. The reason for this wide gap in COX-2 expression in the available studies is difficult to explain, more studies are needed to exclude the possibility of racial factors because most of the available studies are from Asia with few from another continent like Africa. Other possible reasons may include the histological sub-types, tumour grades, tumour stage, study designs, sample size or genetic makeup.

In terms of the histology of CRC, the COX-2 over-expressions in MDA (63.0%) and PDA (55.6%) are higher than WDA (33.3%) and mucinous carcinomas (25%). Similar to this pattern of over-expression, Qi-Bing *et al* [50] in their large-sized study of 1,026 CRC cases in China, showed that the proportions of well, moderate and poorly differentiated CRC cases that over-expressed COX-2 are 73.5%, 80.8% and 74.5%, respectively, while the overall expression is 77.97% with no signiﬁcant correlation with sex, age or tumour location but demonstrate a significant correlation with tumour size ≥5 cm, serosa invasion, late stage and distant metastasis. Likewise, these findings are similar to observations by Yamauchi *et al* [49] in their immunohistochemical analysis of 232 cases of CRC in Japan. Venkatachala and Rajendra [26] on the other hand, observed COX-2 expression in WDA (56.6%), MDA (66.6%) and PDA (100%). The present study demonstrated a strong statistical association between COX-2 expression and histological sub-type of tumour (*p* values of 0.02101) but not with age or sex similar to the findings of large sample size studies of Wu *et al* [50] and Yamauchi *et al* [49]. However, studies with relatively lower sample sizes tend to demonstrate contrasting findings. For instance, findings from the analysis of 30 advanced CRC cases by COX-2 mRNA quantitative PCR, showed that COX-2 mRNA is higher in low-grade CRC (WDA and MDA; 93%) compared to PDA (7%) [51]. Also, two separate studies that analysed 38 and 63 advanced CRC cases by COX-2 IHC showed no significant association between COX-2 expression and histology type and other parameters such as age, sex, tumour size, Duke’s stage, grade, location and depth of invasion [45, 48].

The stage of CRC cases in the studies also affects the rate of COX-2 overexpression. For instance, Venkatachala and Rajendra [26] from India observed overall COX-2 expression in 86.2% of cases. They also observed highly positive COX-2 cases to constitute 33.3% of stage I, 58.8% of stage II, 80% of stage III and 100% of stage IV tumours with association with lymphatic metastasis and invasive depth [26]. Furthermore, the result of COX-2 heterogeneity (varied COX-2 expression among available studies) may also be affected by racial and genetic makeup. Polymorphism in the COX-2 gene with other genes may be key in this regard. Carriers of COX-2 A-1195G AG increase adenomas and CRCs risk, and mutation in the APC gene has been shown to correlate with COX-2 expression in both adenomas and CRCs [11, 52, 53]. More studies are needed to establish the role of race in COX-2 expression in CRCs. 

It is also important to decipher why COX-2 expression is relatively low in many studies. For instance, this current study observed only 46.8% expression despite evidence suggesting COX-2 involvement in every stage of carcinogenesis (initiation and progression) and chemo-preventive effects of NSAIDs [8–10, 50, 54–59]. The supporting evidence includes first, some of the CRCs lacking COX-2 expression over-expressed COX-1 and thus, dysregulation in COX-1 and COX-2 expression is found in a higher percentage of CRCs [60]. Second, any alteration in up- or downstream of COX-2 (arachidonic acid metabolic) pathways may mimic the cellular effect of COX-2. Third, evidence indicates the elevation of COX-2 expression in CRC stromal cells such as fibroblasts and macrophages [61]. Expression of COX-2 is present in both adenomas and CRC because it is believed to be involved in the initiation of tumourigenesis as it can be upregulated by the carcinogen. However, the expression is higher in CRC than in adenoma, indicating that it may also serve as a marker for tumour progression. Expression of COX-2 influences all stages of tumour progression [8–10, 50, 54–59].

## Limitation of this study

The data utilised in this study is limited to northern Nigeria, so multicenter studies in Nigeria and across Africa will give a better reflection of COX-2 expression in CRC in Africa. This will also give a better understanding between COX-2 expression and the uncommon histological subtypes in Africa. The entire tumour had not been stained with COX-2 IHC. Therefore, sections stained with COX-2 antibody may not entirely represent COX-2 status in each CRC case. The inability of this study to correlate COX-2 expression with survival largely due to the loss of patients to follow-up, and the lack of information on the use of COX-2 inhibitors by the patients equally underlies the weakness in this study.

## Conclusion

Occurrence of CRC is more frequent in middle age, and more than half of all cases are early onset CRCs. This trend has clinical, social and financial implications. The strong statistical association between COX-2 over-expression and histological sub-type may influence the outcomes and prevention of CRCs with possible variation in tumour sub-type.

## Conflicts of interest

The authors have declared that no competing interests exist.

## Funding

The authors received no specific funding for this work.

## Figures and Tables

**Table 1.** Shows COX-2 immunoreactive scoring system (IRS).

Intensity (I)		Distribution (D)		IRS (I × D)		COX-2 expression*
Category	score	Category	score	Category	score	Expression
Non	0	Non	0	Negative	0	Negative
Weak	1	1%–10%	1	Weak positive	1–4	Negative
Moderate	2	11%–50%	2	Moderate positive	5–8	Positive
Strong	3	51%–80%	3	Strong positive	9–12	Positive
-	-	>80%	4	-	-	-

* COX-2 over-expression is IRS of 5–12

**Table 2.** Shows age distribution of CRCs with sex, histological subtypes and grade.

Age group (years)									
(%)	10–19	20–29	30–39	40–49	50–59	60–69	70–79	80–89	Total (%)
Sub-type ADC(NOS):WDA	2	3	7	16	12	4	6	1	51 (36.7)
MDA	0	3	8	8	13	8	10	0	50 (36.0)
PDA	0	2	7	3	4	4	0	0	20 (14.3)
									
MAC	0	1	4	4	1	1	1	0	12 (8.6)
SRCC	1	2	0	0	1	1	0	0	5 (3.6)
CCC	0	0	0	0	1	0	0	0	1 (0.7)
									
Total	3	11	26	31	32	18	17	1	139 (100.0)
Grade: Low	2	6	16	23	25	12	16	1	101 (72.7)
High	1	5	10	7	8	6	1	0	38 (27.3)
Total	3	11	26	31	32	18	17	1	139 (100.0)

ADC NOS = Adenocarcinoma not otherwise specified, WDA = Well differentiated adenocarcinoma, MDA = Moderately differentiated adenocarcinoma, PDA = Poorly differentiated adenocarcinoma, SRCC = Signet-ring cell carcinoma, CCC = Clear cell carcinoma and MAC = Mucinous adenocarcinoma

**Table 3.** Shows sex distribution of 139 CRCs by histological subtype and grade.

	Sex		
	Male	Female	Total (%)
Subtype ADC (NOS): WDA MDA PDA MAC SRCC CCC Total Grade: Low High	30 35 12 9 5 1 92 65 27	21 15 8 3 0 0 47 36 11	51( 36.7) 50 (36.0) 20 (14.4) 12 (8.6) 5 (3.6) 1 (0.7) 139 (100.0) 101 (72.7) 38 (27.3)

ADC NOS = Adenocarcinoma not otherwise specified, WDA = Well differentiated adenocarcinoma, MDA = Moderately differentiated adenocarcinoma, PDA = Poorly differentiated adenocarcinoma, SRCC = Signet-ring cell carcinoma, CCC = Clear cell carcinoma and MAC = Mucinous adenocarcinoma

**Table 4.** Shows COX-2 IRS score distribution of 124 CRCs.

Carcinomas	**COX-2 negative		COX-2 positive		Proportion (%)
	IRS (0)	IRS (1–4)	Moderate IRS(5-8)	Strong IRS(9-12)	Percentage of overall positive (%)
**ADC NOS:** **WDA** **MDA** **PDA** **MAC** **SRCC**	10 2 5 3 1	24 15 3 3 0	16 18 5 2 0	1 11 5 0 0	17/51 (33.3) 29/46 (63.0) 10/18 (55.6) 2/8 (25.0) 0
**Total (%)**	21(16.9)	45(36.3)	41(33.1)	17(13.7)	*58/124(46.8)

*Only 124 cases had COX-2 immunohistochemical study, ** COX-2 positive case is defined by an IRS score of 5–12. IRS = immune-reactive score, ADC NOS = Adenocarcinoma not otherwise specified, WDA = Well differentiated adenocarcinoma, MDA = Moderately differentiated adenocarcinoma, PDA = Poorly differentiated adenocarcinoma, SRCC = Signet-ring cell carcinoma, CCC = Clear cell carcinoma and MAC = Mucinous adenocarcinoma

**Table 5.** Shows COX-2 over-expression of 124 CRCs with age group, sex, tumour grade and histological sub-type.

COX-2 over-expression					
Parameters					
		Positivity	Negativity	Total	*p* value
Sex	Male	41	41	82*	0.3144
	Female	17	25	42	
Age group	10–19	0	3	3	0.3578
	20–29	4	4	8	
	30–39	14	11	25	
	40–49	10	16	26	
	50–59	12	16	28	
	60–69	8	10	18	
	70–79	10	5	15	
	80–89	0	1	1	
Grade	Low grade	46	51	97	0.7838
	High grade	12	15	27	
Location	Colon	38	42	80	0.8284
	Rectum	20	24	44	
Type of biopsy	endoscopic	22	27	49	0.082
	Open	36	39	75	
Sub-type					
i-Types	ADC NOS				0.0210*
	WDA	17	34	51	
	MDA	29	17	46	
	PDA	10	8	18	
	MAC	2	6	8	
	SRCC	0	1	1	
	CCC	0	0	0	
ii-MAC versus NMAC)	MAC	2	6	8	0.2493
	NMAC	56	66	122	

* Only 124 out of 139 cases had COX-2 immunohistochemical analysis. ADC NOS = Adenocarcinoma not otherwise specified, WDA = Well differentiated adenocarcinoma, MDA = Moderately differentiated adenocarcinoma, PDA = Poorly differentiated adenocarcinoma, SRCC = Signet-ring cell carcinoma, CCC = Clear cell carcinoma, MAC = Mucinous adenocarcinoma and NMAC = Non mucinous adenocarcinoma

**Table 6.** Shows global studies of COX-2 expression on CRC.

Studies/year	Study period	Country	Cases	Method	Scoring	Stage	COX-2(%)
AFRICA							
Index study	2015–2019	Nigeria	124	IHC	I&D	I~IV	46.8
Kazem *et al* (2013) [22]	2009~2012	Egypt	30	IHC	I&D	II~IV	100
Miladi-Abdennadher *et al* (2012) [62]	-	Tunisia	35	IHC	I&D	I~IV	65.7
ASIA							
Al-Maghrabi *et al* (2012) [63]	2005~2009	Saudi Arabia	94	IHC	I&D	I~IV	56.4
Yoshinaga *et al* (2011) [64]	2003~2007	Japan	52	IHC	1%	I~IV	63.5
Inafuku et al (2009) [65]	1996~2005	Japan	109	IHC	I&D	II	56
Chen *et al* (2008) [66]	1999~2001	China	96	IHC	I&D	I~IV	53.3
Lim *et al* (2008) [67]	1992~2001	Korea	231	IHC	I&D	I~III	42.4
Zhan *et al* (2004) [68]	1992~2001	China	44	IHC	I&D	I~IV	72.7
Soumaoro *et al* (2004) [69]	1986~1996	Japan	288	IHC	I&D	I~IV	70.8
Wu *et al* (2003) [70]	1993~2001	China	139	IHC	I&D	I~IV	84.9
Yamauchi *et al* (2002) [49]	990~1993	Japan	232	IHC	I&D	I~IV	71.6
Joo *et al* (2002) [71]	1995	Korea	60	IHC	I&D	I~IV	70
Tomozawa *et al* (2000) [48]	1990~1994	Japan	63	IHC	I&D	I~III	20.6
Masunaga *et al* (2000) [72]	1990~1999	Japan	100	IHC	I&D	I~IV	72
Europe							
Pancione *et al* (2009) [73]	1999~2009	Italy	72	IHC	50%	I~IV	54.2
Debucquoy *et al* (2009) [74]	1996~2003	Belgium	99	IHC	I&D	NS	80.8
Giralt *et al* (2006) [75]	1994~2001	Spain	74	IHC	I&D	advanced	51.4
Yamac *et al* (2005) [76]	1989~1999	Turkey	83	IHC	I&D	II~IV	62.6
Fux *et al* (2005) [77]	1987~1997	Germany	747	IHC	I&D	I~IV	84.6
Buecher *et al* (2003) [78]	1996~1997	France	61	IHC	5%	I~II	59
Zhang *et al* (2002) [79]	1972~1996	Sweden	112	IHC	10%	I~IV	72.3
Sheehan *et al* (1999) [80]	1988~1991	Ireland	76	IHC	1%	I~IV	81.6
America							
Ogino *et al* (2009) [81]	1976~2002	USA	662	IHC	I&D	I~IV	82.8

IHC = immunohistochemistry, I = intensity of stained cells and D = percentage distribution of tumour cells
